# Aberrant Glymphatic System Function in Chronic Heart Failure with Preserved Ejection Fraction

**DOI:** 10.2174/0115734056497725260909102652

**Published:** 2026-09-11

**Authors:** Jia You, Zongxiang Sheng, Yuting Xia, Yujie Zhang, Chunhua Xing, Huiyou Chen, Yu-Chen Chen, Jinjia Qian, Xindao Yin

**Affiliations:** 1 Department of Radiology, Nanjing First Hospital, Nanjing Medical University, Nanjing, China;; 2 Department of Cardiovascular Ultrasound, Nanjing First Hospital, Nanjing Medical University, Nanjing, China;; 3 Department of Radiology, Nanjing Pukou People's Hospital, Liangjiang Hospital, Southeast University, Nanjing, China

**Keywords:** Chronic heart failure, Preserved ejection fraction, Glymphatic system, Cognitive impairment, Magnetic resonance imaging, HFpEF-related brain injury, Multimodal neuroimaging

## Abstract

**Background:**

Cognitive dysfunction is frequently observed in chronic Heart Failure with preserved Ejection Fraction (HFpEF) patients, while the underlying neuropathological basis remains incompletely characterized. This study aimed to demonstrate aberrant glymphatic system function in chronic HFpEF and its association with cognitive ability.

**Materials and Methods:**

We proposed a multimodal neuroimaging model combining the Analysis of Perivascular Space Index (ALPS-index), Choroid Plexus Volume (CPV), and Perivascular Space (PVS) for early detection of HFpEF-related brain injury. Thirty-one chronic HFpEF patients and 32 Healthy Controls (HCs) were enrolled and underwent brain MRI and cognitive function tests. Associations between glymphatic markers, cognitive scores, and cardiac function were analyzed.

**Results:**

The ALPS-index in patients with chronic HFpEF was significantly decreased (*p* = 0.017), and the volumes of CPV and PVS were larger (*p* = 0.004; *p* = 0.011). Furthermore, correlation analysis revealed a significant positive correlation between CPV and TMT-B scores (r = 0.634, *p* < 0.001), and CPV showed an inverse correlation with Left Ventricular Ejection Fraction (LVEF) (r = -0.542, *p* = 0.003) and Fractional Shortening (FS) (r = -0.508, *p* = 0.006). The diagnostic efficacy of the multimodal model (ALPS-index + CPV + PVS) was improved compared to a single marker (AUC = 0.79 *vs*. AUC = 0.75 for CPV alone).

**Discussion:**

These findings suggest that chronic HFpEF is associated with glymphatic dysfunction, as reflected by a reduced ALPS-index and enlarged CPV and PVS. The correlation between CPV and both cognitive and cardiac parameters highlights a potential heart–brain axis mechanism. The multimodal imaging approach demonstrated promising diagnostic value, supporting its potential utility for early detection of cerebral involvement in HFpEF.

**Conclusion:**

Chronic HFpEF may induce dysfunction of the glymphatic system, which is manifested as decreased ALPS-index, enlarged CPV and PVS, and is associated with cognitive decline and cardiac dysfunction. Multimodal neuroimaging markers may serve as promising neuroimaging markers for the early identification of chronic HFpEF-related cognitive impairment.

## 1 INTRODUCTION

Chronic Heart Failure (HF) refers to a complex clinical syndrome that arises from abnormal heart structure or function; the heart cannot pump blood efficiently enough over a prolonged period to meet the metabolic needs of the body, subsequently resulting in corresponding symptoms and signs [1]. Based on the measurement results of Left Ventricular Ejection Fraction (LVEF), HF mainly presents in three different types: Heart Failure with reduced Ejection Fraction (HFrEF), Heart Failure with preserved Ejection Fraction (HFpEF), and heart failure with mid-range ejection fraction. Almost half of the patients with HF belong to the HFpEF category. At present, the prevalence of HF is constantly increasing as a result of the aging population, improvement in the treatment of ischemic heart disease, and an increase in patient survival rates [2]. However, long-term HF affects not only the heart itself but also other organ systems, such as the brain [3, 4]. In recent years, the damage to the central nervous system caused by chronic HF has gradually attracted wide attention. Some studies have shown that about 25%-50% of all HF patients have varying degrees of cognitive deficits. The prevalence of dementia is about 10%-20%, which is 2-3 times that of individuals without HF of the same age [5, 6]. Therefore, it is very important to further explore early cognitive deficits and the mechanisms underlying their progression in patients with chronic HFpEF.

Currently, the mechanisms underlying neurological impairment associated with chronic HFpEF have not been fully determined. Contributing factors may include disorders of cerebral blood perfusion, neuroendocrine activation, neuroin-flammation, and oxidative stress [7, 8]. The most effective strategy for managing cognitive impairment lies in early detection and prevention of these neurological impairments. Identifying reliable biomarkers for the early prediction of cognitive impairment is crucial for preventing disease progres-sion and poor cognitive outcomes. A recent resting-state functional Magnetic Resonance Imaging (rs-fMRI) study has clearly demonstrated that HFpEF patients exhibit abnormal static and dynamic functional network connections, providing direct evidence for the association between this disease and cognitive dysfunction [9]. In addition to functional abnormalities, chronic HF patients also show significant alterations in brain structure, such as gray matter atrophy and an increase in white matter lesions [10-12]. Gray-matter atrophy in HF patients is associated with impairment of cognitive function, particularly memory formation and learning capacity [11, 13]. Chronic HF may lead to cerebral hypoperfusion, which can induce a series of neurophysiological alterations due to insufficient blood supply. This inadequate blood flow may result in neuronal damage and apoptosis, potentially causing long-term irreversible damage to brain structure [14]. Beyond macrostructural changes, recent research has shifted focus toward impaired cerebral clearance mechanisms.

With the development of neuroimaging technology, the Glymphatic System (GS) has attracted researchers’ attention for the study of neurodegenerative diseases [15]. The GS structure, formed between the foot processes of astrocytes and the vascular wall, functions to remove cellular metabolic waste [16]. When the system malfunctions, abnormal proteins such as beta-amyloid and tau proteins accumulate in the brain [17]. It is noteworthy that HF patients usually have chronic cerebral hypoperfusion and systemic inflammation, which can damage glymphatic function [18, 19]. Previous studies have found that HF can disrupt the homeostasis of the glymphatic system, manifested as an increase in the inflow of cerebrospinal fluid into brain tissue, but the brain's ability to clear waste does not improve simultaneously, leading to an imbalance in the fluid dynamics of brain tissue. This kind of glymphatic dysfunction is closely related to the reduction of cerebral blood flow and may be one of the important mechanisms of cognitive impairment associated with HF [18]. Meanwhile, other research has indicated that after HF, cardiac-derived extracellular vesicles can cross the blood-brain barrier and accumulate in key cognitive regions such as the hippocampus and cortex, and be taken up by microglia, triggering neuroinflammatory responses [20]. During this process, the glymphatic system, as the main metabolic waste clearance pathway of the brain, may play a crucial role. Under neuroinflammatory conditions, microglia become activated and inflammatory factors are released, which may affect the functional efficiency of the glymphatic system, further accelerating the accumulation of metabolic waste in the brain and accelerating cognitive decline. Additionally, research findings suggest that HF is associated with dysfunction in cerebral autoregulation and changes in blood-brain barrier permeability. These alterations may hinder glymphatic flow and diminish the effectiveness of metabolic clearance [21, 22]. Therefore, studying changes in the glymphatic system in HF patients may not only open new paths to explore the causes of cognitive impairment but also provide new potential biomarkers for early diagnosis and prevention. Imaging biomarkers such as the Analysis Along the Perivascular Space Index (ALPS-index), Choroid Plexus Volume (CPV), and Perivascular Space Volume (PVS) have been developed to indirectly assess glymphatic system function. Relevant studies have shown that, for patients with Atrial Fibrillation (AF), increases in the ALPS-index are observed after catheter ablation surgery, indicating that the restored hemodynamics may, to some extent, reverse disruption of cerebrospinal fluid circulation [23]. CPV enlargement is linked to dysregulated cerebrospinal fluid production and neuroinflammation [24]. In contrast, PVS enlargement is considered a marker of impaired perivascular clearance and is associated with various neurological conditions [25]. Together, these non-invasive markers provide a multidimensional window into glymphatic activity *in vivo.*

Based on the above research background, we hypothesized that chronic HFpEF may result in dysfunction of the brain’s glymphatic system. These alterations may be associated with cognitive decline. Moreover, a combined multimodal biomarker approach may offer superior diagnostic performance compared to any single biomarker. This approach provides a new strategy for early detection of cerebral abnormalities in chronic HFpEF.

## 2 MATERIALS AND METHODS

### 2.1 Subjects and Clinical Data

The Research Ethics Committee at Nanjing First Hospital has granted approval for this study. Prior to the commencement of the research, all participants provided their written informed consent. Patients with clinically confirmed chronic HFpEF were included. The criteria used for diagnosing HF are the particular benchmarks established in the guidelines provided by the European Society of Cardiology [26]. Echocardiography was required to demonstrate normal or mildly impaired LVEF, with reduced LVEF defined as <50%. From October 2022 to June 2025, we recruited a cohort consisting of 31 patients diagnosed with chronic HFpEF and 32 healthy control participants, who were appropriately matched based on age, gender, and educational background. Inclusion criteria are as follows: (a) age of 18 years or above, with no upper age limit; (b) confirmed chronic HFpEF diagnosis; (c) no history of psychiatric disorders or alcohol use disorder; (d) no liver cirrhosis, hepatic encephalopathy, thyroid dysfunction, cerebrovascular disease, or any other diseases that may affect cognitive function; (e) no history of drug abuse or dependence, and during the assessment period, no sedative-hypnotic or antidepressant drugs were used, and no long-term use of cognitive-enhancing drugs. Exclusion criteria are as follows: (a) hearing, reading, language expression, or writing disorders; (b) severe heart, liver, lung, kidney, and hematopoietic system diseases; (c) history of neuropathy such as stroke, Parkinson's Disease (PD), epilepsy, Alzheimer's Disease (AD), etc.; (d) history of mental disorders including depression, mania, and alcohol dependence; (e) history of loss of consciousness caused by neurological diseases and brain trauma; (f) any implant or device impeding MRI.

The demographic characteristics were collected for all participants. LVEF, Fractional Shortening (FS), Left Ventricular End-diastolic Diameter (LVDd), and Left Ventricular End-systolic Diameter (LVDs) were measured by echocardiography in the HFpEF group.

### 2.2 Cognitive Assessment

A series of cognitive assessments were carried out in accordance with established protocols to evaluate various dimensions of cognitive functioning. The assessments specifically emphasized memory, attention, and executive functions. Assessments were administered to all individuals involved, including the following tests: Mini-Mental State Exam (MMSE) [27] and Montreal Cognitive Assessment (MoCA) [28] for the global assessment of cognitive status, including assessments of language abilities and cognitive reserve; Auditory Verbal Learning Test (AVLT) [29] and Complex Figure Test (CFT) [30] for verbal episodic memory learning and visual memory recall; Digit Span Test (DST) [31] for working memory capacity. In addition, attention and executive functions were evaluated using the Trail Making Test (TMT) [32] and Clock-Drawing Test (CDT) [33]. The TMT consists of two parts: TMT-A primarily evaluates motor speed and visual scanning abilities, whereas TMT-B is used to measure cognitive flexibility. Moreover, the Digit Symbol Substitution Test (DSST) [34] and the Verbal Fluency Test (VFT) [35] were employed to assess both cognitive processing speed and visuospatial skills. On average, each participant required approximately 50 minutes to complete all assessments.

### 2.3 Imaging Procedure

All participants underwent functional magnetic resonance imaging scans on a 3.0T MRI scanner (produced by Philips Medical Systems) using an eight-channel digital head coil with parallel imaging capabilities. Participants were advised to relax, keep their eyes closed, and remain still throughout the scan. For each research subject, Diffusion Tensor Imaging (DTI) data and high-resolution T1-weighted anatomical data were acquired. The MRI acquisition protocol included the following steps: (a) DTI parameters were as follows: TR = 3000 ms; TE = 100 ms; thickness = 2.5 mm; slices = 55; flip angle (FA) = 90°; gap = 0; Field of View (FOV) = 240 mm × 240 mm; acquisition matrix = 120 × 177; Thirty-two volumes with diffusion gradients along 32 nonlinear directions (b = 1000 s/mm^2^) were acquired. Additionally, one non-diffusion-weighted volume (b = 0 s/mm^2^) was acquired. The total acquisition time of the DTI sequence was 11 minutes and 9 seconds; (b) high-resolution anatomical images were obtained using the T1-weighted imaging sequence with Three-dimensional Turbo Rapid Echo (3D-TFE). The parameters were as follows: TR = 8.1 ms; TE = 3.7 ms; thickness = 1 mm; slices = 172; FA = 8°; gap = 0; FOV = 256 mm × 256 mm; acquisition matrix = 256 × 256. The total duration of the entire structure scan was 5 minutes and 28 seconds.

### 2.4 ALPS-index Processing and Measurement

The initial MRI data for every participant were preserved in the Digital Imaging and Communications in Medicine (DICOM) format. In order to streamline subsequent analysis, we used MRIcron for the conversion of DICOM files to the NIFTI format, thus ensuring data consistency and compatibility. Subsequently, the data were subjected to a multi-step preprocessing using the FMRIB Software Library (FSL, http://www.fmrib.ox.ac.uk/fsl). This preprocessing included noise filtering, correction for magnetic resonance artifacts, as well as image distortion caused by motion, and reduction of Gibbs ringing artifacts [36]. Throughout the process, motion parameters were estimated. No participants were eliminated from the study on the grounds of excessive head movement (more than 2 standard deviations above the average). Next, the head-extraction technique was employed to remove non-brain tissues, thereby enhancing the accuracy of the analysis. The generation of the DTI parameter map was performed using the FSL diffusion toolbox. This toolbox calculates fractional anisotropy, which quantifies the directional dependence of diffusion, along with additional metrics such as eigenvectors (V1, V2, V3). These eigenvectors represent the orientations of fundamental brain constituents, including neural fibers and vascular structures.

The process of calculating the ALPS-index involves the following steps. Firstly, a rectangular Region of Interest (ROI) was defined. Next, fiber directions and diffusion rates along the x-, y-, and z-axes were extracted for each voxel layer within this ROI (Fig. **1A**). From this collection of voxels, we identified an individual ROI for each fiber, aligned along the same x-axis (specifically, the projected fiber and its corresponding fiber), that exhibited the greatest directional consistency within that fiber. Finally, the ALPS-index was calculated using the following formula: ALPS-index =mean (D_x,proj_, D_x,assoc_)/ mean (D_y,proj_, D_z,assoc_). D_x,proj_: diffusivity along the x-axis in the projection fiber, D_x,assoc_: diffusivity along the x-axis in the association fiber, D_y,proj_: diffusivity along the y-axis in the projection fiber, D_z,assoc_: diffusivity along the z-axis in the association fiber.

When the movement of water through the PVS is limited or significantly hindered, the ALPS-index typically nears the value of 1. Conversely, a higher ALPS-index indicates stronger water mobility within the PVS, suggesting better glymphatic function [37, 38].

### 2.5 CPV and PVS Volume Measurement

To measure the volumes of the CPV and PVS, we used the convolutional neural network framework developed by the Yazdan-Panah group for segmentation and quantification of the CPV (https://github.com/hettk/chp_seg). The accuracy of this model is higher than that of other methods, such as FreeSurfer [39]. The final mask for the choroid plexus was created based on the T1-weighted images. For more methodological details, please refer to the original paper [39]. Subsequently, two neuroradiologists, who were completely unaware of the clinical data of the subjects, re-examined all automatically segmented choroid plexus images and made manual corrections. To segment the PVS, we utilized an already-developed deep learning model that produced probability maps for the PVS from T1-weighted imaging data [37]. Based on the recommendations provided by the developer and visual assessments, a probability threshold of 0.5 was established for the creation of PVS masks. Following a thorough manual refinement process, the volumes of the CPV and PVS were individually computed in preparation for subsequent statistical evaluation. The Total Intracranial Volume (TIV) was also calculated and used as a covariate. Figure **1B** and **C** illustrate the segmentation process of the CPV and PVS volumes.

### 2.6 Statistical Analysis

The demographic and clinical characteristics were analyzed using SPSS (version 19.0 for Windows). An independent-samples t-test was used to compare age, educational level, all cognitive scores, TIV, ALPS-index, CPV, and PVS among the different groups. Gender was compared between the two groups using the Chi-squared test. Age, gender, educational level, and TIV are regarded as relevant variables. The statistical significance was set at *p* < 0.05. Given the exploratory nature of this study, we set the statistical significance threshold at *p* < 0.05 (two-tailed) without adjustment for multiple comparisons. To further assess the robustness of our main findings, post-hoc Bonferroni corrections were planned. Results with marginal significance after correction were interpreted with caution.

To evaluate correlations between glymphatic parameters (ALPS-index, CPV, PVS) and echocardiographic parameters as well as cognitive scores, a Pearson correlation analysis was performed separately for each item. Age, gender, educational level, as well as TIV are regarded as relevant variables. The statistical significance was set at *p* < 0.05.

The diagnostic performance of each glymphatic biomarker (ALPS-index, CPV, PVS volume) was initially evaluated using a Receiver Operating Characteristic (ROC) curve; the Area Under the Curve (AUC) as well as the corresponding 95% confidence interval were calculated. The optimal cut-off value was determined by maximizing the Youden index. In order to assess the integrated diagnostic value of these markers, a binary logistic regression model was developed. This model incorporated the ALPS-index, CPV and PVS volume. Multicollinearity was excluded from consideration, as indicated by a variance inflation factor of less than 5 for each predictor variable. Subsequently, the model's predicted probabilities were used to construct a comprehensive ROC curve.

## 3 RESULTS

### 3.1 Demographics and Clinical Characteristics

Table **1** provides an overview of the demographic and clinical information. The HFpEF and HCs groups were well-matched in terms of age, gender, and educational level (all *p* > 0.05). In both the CFT and CFT-delayed scoring assessments, individuals in the HFpEF group exhibited significantly lower scores than those in the HC group (all *p* < 0.05; Table **1**). Additionally, in the TMT-B evaluation, the HFpEF group showed a significantly higher score than the HC group (*p* < 0.05, Table **1**). There were no statistically significant differences in other assessed cognitive domains between the two groups.

### 3.2 ALPS-index, CPV and PVS Differences

Figure **2** and Table **2** summarize the comparison of biomarkers of the glymphatic system. There are significant differences between the HCs group and the HFpEF group in terms of the ALPS-index, CPV, and PVS. Compared with the HCs group, the ALPS-index of patients with HFpEF was significantly lower (t = 2.465, *p* = 0.017). Conversely, in the HFpEF group, the volumes of CPV and PVS were significantly larger than those in the HCs group (CPV: t = -2.972, *p* = 0.004; PVS: t = -2.670, *p* = 0.011).

### 3.3 Correlation Analysis

In HFpEF patients, a significant positive correlation was found between CPV and TMT-B score (r = 0.634, *p* < 0.001) (Fig. **3A**). Furthermore, CPV was negatively correlated with LVEF (r = -0.542, *p* = 0.003) (Fig. **3B**). CPV was also negatively correlated with FS (r = -0.508, *p* = 0.006) (Fig. **3C**). However, the ALPS-index and PVS showed no significant correlations with the other measured parameters.

### 3.4 Diagnostic Performance

ROC curve analysis demonstrated varying levels of diagnostic performance across the glymphatic biomarkers evaluated (Fig. **4**). Among all the indicators, the CPV has the strongest discriminatory ability (AUC = 0.75, 95% confidence interval: 0.62-0.87, *p* = 0.0007), followed by the ALPS-index (AUC = 0.67, 95% confidence interval: 0.53-0.80, *p* = 0.0242) and PVS (AUC = 0.62, 95% confidence interval: 0.48-0.77, *p* = 0.0990). Moreover, it is worth noting that the comprehensive model (including CPV, ALPS-index, and PVS) showed promising diagnostic performance (AUC = 0.79, 95% confidence interval: 0.68-0.90, *p* < 0.0001).

## 4 DISCUSSION

In patients with HFpEF, we observed decreased glymphatic activity. In particular, the ALPS index, which serves as an indicator of perivascular glymphatic activity, was found to be markedly reduced in patients with HFpEF compared to matched healthy controls. Additionally, the CPV and PVS were increased in HFpEF patients. Moreover, CPV was positively correlated with the TMT-B score and negatively correlated with LVEF and FS. Furthermore, a combined model incorporating the ALPS-index, CPV, and PVS showed promising diagnostic accuracy in detecting brain changes in HFpEF patients. To the best of our knowledge, this is the first exploratory study to demonstrate the association between impaired glymphatic function and cognitive impairment in HFpEF patients. This study provides preliminary evidence elucidating the underlying neuropathological mechanism contributing to cognitive deficits in chronic HFpEF.

The glymphatic system relies on the processes of normal cerebrospinal fluid flow and waste clearance within tissues. However, these processes may be hindered due to chronic cerebral hypoperfusion and neuroinflammatory responses associated with HF [18]. Previous studies have shown that the ALPS-index can serve as an effective indicator for measuring glymphatic function. A low ALPS-index value is associated with an increased risk of cognitive decline in patients with HF [14, 40]. The diminished ALPS-index identified in our investigation may indicate impaired water movement along perivascular channels. This impairment leads to glymphatic inefficiency and subsequent neural damage. Similar pathways have also been reported in AD and PD, particularly demonstrated by the well-established inverse relationship between the ALPS-index and cognitive performance [41, 42]. Therefore, chronic HF can impair glymphatic function through various mechanisms, including insufficient cerebral perfusion, vascular lesions, and accumulation of toxic proteins. We propose that a reduction in the ALPS-index can serve as a biomarker for assessing neural damage associated with glymphatic dysfunction.

Notably, CPV emerged as a robust biomarker in our study, showing significant correlations with both cognitive performance and cardiac function. The TMT-B is a classic test of executive function, where a longer duration (higher score) indicates poorer performance. The positive association between CPV and TMT-B scores suggests that choroid plexus enlargement may be linked to poorer executive function. This association may result from the choroid plexus’s dual functions in cerebrospinal Fluid (CSF) secretion and immune regulatory functions related to the blood-brain barrier, with its volume expansion altering CSF dynamics and promoting the diffusion of neuroinflammatory mediators [43]. Furthermore, the negative correlation between CPV and LVEF/FS indicates the interaction between cardiac dysfunction and central nervous system alterations, suggesting that the choroid plexus may be a potential mediator factor for brain damage associated with heart failure [44]. These results are consistent with findings from studies on the role of the choroid plexus in neuroinflammation and neurodegenerative diseases, and offer a new perspective for comprehending the pathological mechanisms of the “heart-brain axis”. They suggest that the choroid plexus may serve as a potential anatomical hub linking cardiac dysfunction to neurodegenerative diseases [45].

In chronic HFpEF patients, enlarged PVS may indicate perivascular dysfunction. Although our study did not find a direct correlation between PVS and cognitive or cardiac parameters, the increased prevalence of enlarged PVS in chronic HFpEF patients suggests broader impairment of cerebral waste-clearance pathways. This dysfunction could result in the buildup of neurotoxic byproducts, including beta-amyloid protein, which may subsequently increase the likelihood of cognitive deterioration in individuals with chronic HF [22]. Previous research supports this link by demonstrating that glymphatic dysfunction is closely associated with pathological protein accumulation in neurodegenerative disorders like AD [42, 46]. These results suggest that the enlarged PVS may serve as a potential imaging indicator of impaired waste clearance function in patients with chronic HFpEF.

The promising diagnostic performance of the combined model, although derived from an exploratory analysis with a modest sample size, highlights the clinical potential of multimodal neuroimaging biomarkers for detecting early cerebral alterations in chronic HFpEF patients. These preliminary findings warrant further validation in larger independent cohorts. The integration of these neuroimaging markers may enable a more comprehensive assessment of glymphatic dysfunction, thereby encompassing various aspects of its pathophysiological process. Such multimodal assessment may prove particularly valuable for early identification of patients with chronic HFpEF at risk of cognitive decline, enabling timely therapeutic interventions.

## 5 STUDY LIMITATIONS

There are several limitations in the current study. First, the cross-sectional nature of this study precludes the determination of causal relationships. It is essential to conduct a prospective longitudinal study to illustrate the temporal changes in glymphatic dysfunction during the progression of HF. Second, the limited sample size may affect the external validity and statistical power of our conclusions. Therefore, more extensive, multi-center cohort studies are required to confirm and generalize these results. Third, although we controlled for key covariates, unmeasured confounders (*e.g*., sleep disorders or medication effects) may still influence glymphatic function. Fourth, echocardiographic parameters were not available for the healthy control group, as these examinations were not part of the routine clinical protocol for healthy volunteers; this limits the direct comparison of cardiac structural and functional profiles between the two groups, and we will consider including such data in our future studies. Fifth, the lack of adjustment for multiple comparisons represents a limitation of this exploratory study. Post-hoc Bonferroni corrections confirmed the robustness of most core findings (CPV, PVS, and the correlations involving CPV), while the ALPS-index result was marginally non-significant after correction (raw p = 0.017 *vs*. corrected α = 0.0167), suggesting this finding should be interpreted cautiously and requires future validation. Future studies will continue to address these limitations and gradually improve the understanding of the mechanism underlying the heart-brain axis.

## CONCLUSION

In summary, this exploratory study has revealed that chronic HFpEF patients show significant abnormalities in the glymphatic system, characterized by a decreased ALPS-index and increased CPV and PVS. These changes are associated with cognitive dysfunction and cardiac dysfunction, suggesting that HF may cause brain injury through some mechanism. Using biomarkers related to the glymphatic system for detection, this approach may serve as a potential non-invasive method for early identification and tracking of neurological dysfunction associated with HF. Future research should explore the long-term development of these changes and their therapeutic significance, thereby paving the way for the development of targeted intervention measures to protect the cognitive function of patients with chronic HF.

## Figures and Tables

**Fig. (1):**
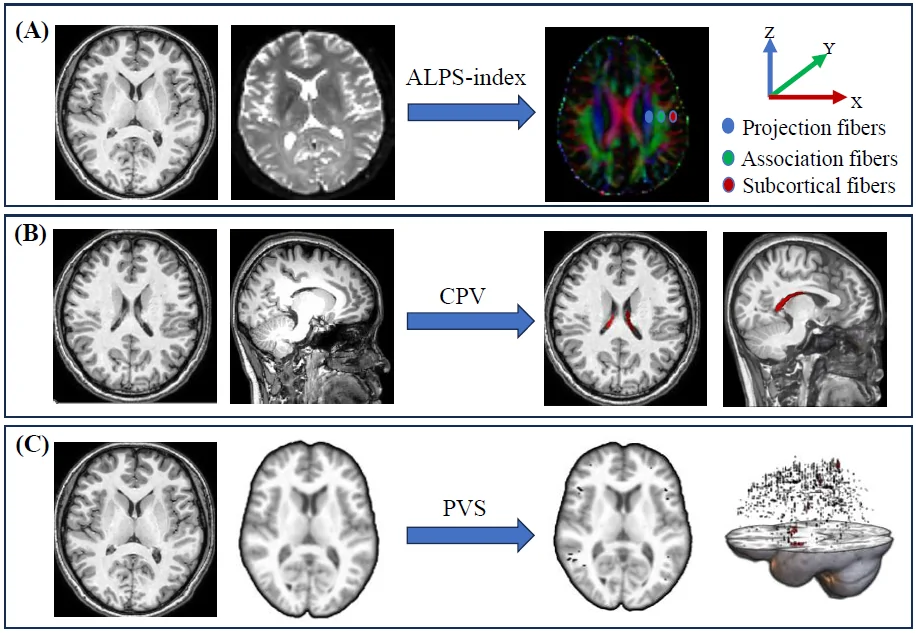
ALPS-index, CPV and PVS measurement methods. (**A**) Illustration of the ALPS-index calculation process, which involves DTI fitting and ROI placement. Projection fibers (blue), association fibers (green), and subcortical fibers (red) are visualized along the z-axis, y-axis, and x-axis, respectively. CPV (**B**) and PVS (**C**) were segmented from T1-weighted images using a convolutional neural network and a deep learning model, respectively. **Abbreviations:** ALPS: analysis along the perivascular space; CPV: choroid plexus volume; PVS: perivascular space; DTI: diffusion tensor image; ROI: region of interest.

**Fig. (2):**
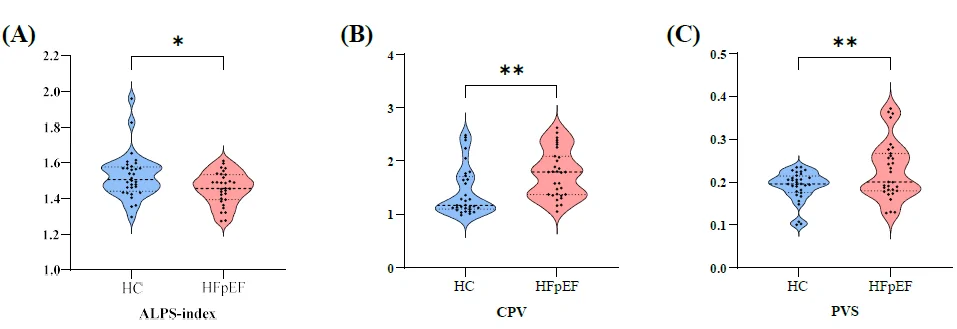
Violin plots of group differences of ALPS-index (**A**), CPV (**B**), and PVS (**C**) in HCs and HFpEF patients. **Abbreviations:** ALPS: analysis along the perivascular space; CPV: choroid plexus volume; PVS: perivascular space; HC: healthy controls; HFpEF: heart failure with preserved ejection fraction.

**Fig. (3):**
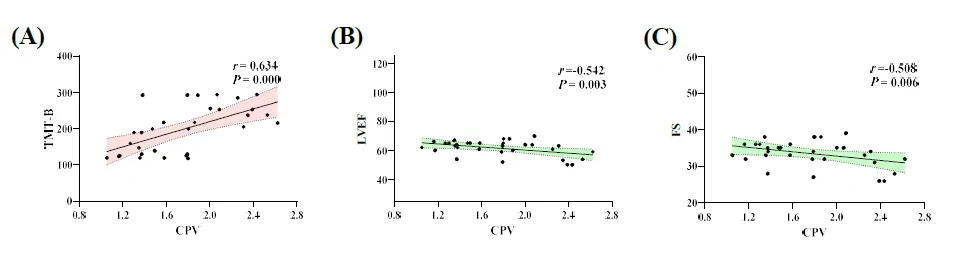
Correlations among CPV measurements, cognitive scores (TMT-B), and echocardiographic parameters (LVEF, FS) in patients. CPV showed a positive correlation with TMT-B scores but negative correlations with LVEF and FS. **Abbreviations:** CPV: choroid plexus volume; TMT-B: Trail Making Test-Part B; LVEF: left ventricular ejection fraction; FS: fractional shortening.

**Fig. (4):**
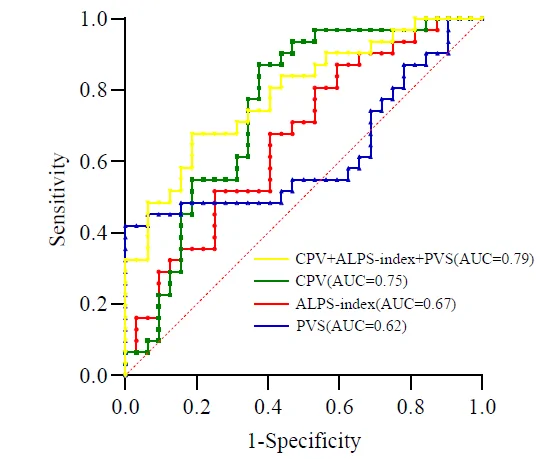
Diagnostic performance of glymphatic biomarkers for HFpEF patients. ROC curves for CPV (AUC = 0.75), ALPS-index (AUC = 0.67), PVS (AUC = 0.62), and their combined model (AUC = 0.79). **Abbreviations:** HFpEF: heart failure with preserved ejection fraction; ROC: receiver operating characteristic; CPV: choroid plexus volume; AUC: area under the curve; ALPS: analysis along the perivascular space; PVS: perivascular space.

**Table 1:** Demographic and clinical characteristics in patients with HCs and HF.

Characteristics	HCs (n = 32)	HF (n = 31)	t/χ^2^ value	*P*-value
Age (years)	55.97±8.98	59.39±6.68	-1.710	0.092
Gender (male/female)	18/14	18/13	0.021	0.543
Education	11.56±2.12	12.10±2.32	-0.955	0.343
MMSE	29.09±0.86	28.61±1.26	1.780	0.080
MoCA	27.19±1.64	27.16±1.88	0.059	0.953
VFT	14.41±4.06	14.77±3.77	-0.372	0.711
CFT	34.69±1.44	31.53±7.37	2.341	0.026*
CFT-delay	21.13±6.40	17.03±3.09	3.245	0.002*
TMT-A	68.75±20.16	65.55±17.30	0.676	0.502
TMT-B	166.50±41.48	201.03±63.13	-2.557	0.014*
AVLT	32.31±8.85	34.81±8.07	-1.168	0.247
AVLT-delay	5.97±2.75	6.68±2.61	-1.047	0.299
CDT	3.66±0.55	3.58±0.56	0.541	0.591
DST	11.63±1.68	11.61±1.98	0.026	0.979
DSST TIV LVEF FS (%) LVDd (mm) LVDs (mm)	69.47±10.61 1373.04±109.24	67.61±10.78 1370.23±123.65 61.52±5.37 33.48±3.56 48.29±5.21 31.94±4.55	0.689 0.096	0.494 0.924

**Note:** Data are the mean ± standard deviation.

**Abbreviations:** HCs: healthy controls; HF: heart failure; MMSE: Mini-Mental State Examination; MoCA: Montreal Cognitive Assessment; VFT: Verbal Fluency Test; CFT: Complex Figure Test; TMT-A: Trail Making Test-Part A; TMT-B: Trail Making Test-Part B; AVLT: Auditory Verbal Learning Test; CDT: Clock Drawing Test; DST: Digit Span Test; DSST: Digit Symbol Substitution Test; TIV: total intracranial volume; LVEF: Left ventricular ejection fraction; FS: fractional shortening; LVDd: left ventricular end-diastolic diameter; LVDs: left ventricular end-systolic diameter.

*P < 0.05.

**Table 2:** Comparison of ALPS-index, CPV and PVS between HCs and HF.

-	**HCs (n=32)**	**HF (n=31)**	**t-value**	***P-*value**
ALPS-index	1.53±0.13	1.46±0.09	2.465	0.017*
CPV	1.42±0.46	1.76±0.45	-2.972	0.004*
PVS	0.19±0.04	0.23±0.07	-2.670	0.011*

**Note:** Data are the mean ± standard deviation.

**Abbreviations:** HCs: healthy controls; HF: heart failure; ALPS: analysis along the perivascular space; CPV: choroid plexus volume; PVS: perivascular space.

*P < 0.05.

## Data Availability

The datasets used and analyzed during the current study are available from the corresponding authors [X.Y.] and [J.Q.] on reasonable request.

## LIST OF ABBREVIATIONS

HF: Heart Failure
LVEF: Left Ventricular Ejection Fraction
HFrEF: Heart Failure with Reduced Ejection Fraction
HFpEF: Heart Failure with Preserved Ejection Fraction
rs-fMRI: Resting-State Functional Magnetic Resonance Imaging
GS: Glymphatic System
ALPS-index: Analysis Along the Perivascular Space Index
CPV: Choroid Plexus Volume
PVS: Perivascular Space Volume
AF: Atrial Fibrillation
PD: Parkinson's Disease
AD: Alzheimer's Disease
FS: Fractional Shortening
LVDd: Left Ventricular End-Diastolic Diameter
LVDs: Left Ventricular End-Systolic Diameter
MMSE: Mini-Mental State Exam
MoCA: Montreal Cognitive Assessment
AVLT: Auditory Verbal Learning Test
CFT: Complex Figure Test
DST: Digit Span Test
TMT: Trail Making Test
CDT: Clock-Drawing Test
DSST: Digit Symbol Substitution Test
VFT: Verbal Fluency Test
DTI: Diffusion Tensor Imaging
FA: Flip Angle
FOV: Field of View
3D-TFE: Three-Dimensional Turbo Fast Echo
DICOM: Digital Imaging and Communications in Medicine
ROI: Region of Interest
TIV: Total Intracranial Volume
ROC: Receiver Operating Characteristic
AUC: Area under the Curve
CSF: Cerebrospinal Fluid

## STANDARDS OF REPORTING

STROBE guidelines were followed.
